# Zoledronic acid and teriparatide have a complementary therapeutic effect on aseptic loosening in a rabbit model

**DOI:** 10.1186/s12891-021-04458-4

**Published:** 2021-06-24

**Authors:** Peng Wang, Guang-qian Shang, Shuai Xiang, Hai-ning Zhang, Ying-zhen Wang, Hao Xu

**Affiliations:** Department of Joint Surgery, The Affiliated Hospital of Qingdao University, Qingdao, 266000 Shandong China

**Keywords:** Aseptic loosening, Wear debris, Osteoblast, Osteoclast, Zoledronic acid, Teriparatide

## Abstract

**Background:**

Revisions are mainly caused by wear debris-induced aseptic loosening. How to effectively suppress debris-induced periprosthetic osteolysis has become an urgent problem. Both zoledronic acid and teriparatide can increase the bone mass around prostheses and increase the stability of prostheses. A hypothesis was proposed: the combination of the two drugs may have a better treatment effect than the use of either drug alone.

**Methods:**

We created a rabbit model to study the effect and mechanism of the combination of zoledronic acid and teriparatide in the treatment of aseptic loosening. Thirty-two adult male New Zealand white rabbits were selected and treated with TKA surgery, and a titanium rod prosthesis coated evenly with micrometre-sized titanium debris was implanted into the right femoral medullary cavity. All rabbits were randomized into four groups (control group = 8, zoledronic acid group = 8, teriparatide group = 8, and zoledronic acid + teriparatide group = 8). All the animals were sacrificed in the 12th week, and X-ray analyses, H&E staining, Goldner-Masson trichrome staining, von Kossa staining, and RT-PCR and Western blotting of the mRNA and protein of OCN, OPG, RANKL and TRAP5b in the interface membrane tissues around the prostheses were immediately carried out.

**Results:**

The results shown that both zoledronic acid and teriparatide could inhibit debris-induced peri-prosthetic osteolysis and promote new bone formation. Zoledronic acid was more capable of inhibiting osteoclast activation and peri-prosthetic osteolysis, while teriparatide was more capable of promoting osteoblast function and peri-prosthetic bone integration.

**Conclusion:**

This research confirmed that the combination of zoledronic acid and teriparatide could prevent and treat aseptic loosening of the prosthesis more effectively. However, the safety of this combination and the feasibility of long-term application have not been ensured, and the clinical application requires further experiments and clinical research support.

**Supplementary Information:**

The online version contains supplementary material available at 10.1186/s12891-021-04458-4.

## Introduction

With the maturity and wide application of artificial joint replacement for more than 20 years, prolonging the service life of joint prostheses has become the most concerning topic for orthopaedists. More than 2/3 of revisions are caused by aseptic loosening [1], and approximately 50% of cases of aseptic loosening are induced by wear debris [2]. In the clinic, many patients with peri-prosthetic osteolysis are asymptomatic, even though osteolysis is already apparent on imaging [3, 4]. The corresponding clinical manifestations appear only when osteolysis is serious enough to damage the stability of the prosthesis. Revision surgery performed for these patients often results in unimaginable large bone defects. Compared with primary surgery, revision surgery is more difficult, more traumatic, and less effective, and it results in a shorter service life of the joint prosthesis [5]. Therefore, effectively suppressing debris-induced periprosthetic osteolysis has become an urgent problem.

The debris-induced biological response is considered the primary cause of periprosthetic osteolysis and aseptic loosening [6]. According to domestic and foreign research results, particle-induced osteoclast activation plays a key role in the course of aseptic loosening [7–9]. Osteoclast activation can be induced by a variety of cytokines, such as TNF-α, IL-1 and IL-6. Under the stimulation of particulate debris, cytokines can be secreted by a variety of essential human cells, such as macrophages, fibroblasts, foreign body giant cells, and osteoblasts [10]. These cytokines directly enhance the bone resorption function of osteoclasts around the prosthesis and create a gap between the prosthesis and bone, resulting in loosening of the artificial joint prosthesis. These cytokines also stimulate pro-osteoclast and blood-derived macrophages to transform into osteoclasts or osteoclast-like cells and ultimately increase bone resorption. In short, the most critical mechanism of prosthesis loosening is the synergistic effects of increased peri-prosthetic bone absorption and suppressed new bone formation [11].

Bisphosphonates and parathyroid hormones (PTHs) are effective drugs that can promote new bone formation and suppress bone resorption and are extensively applied in the treatment of osteoporosis. Zoledronic acid, as a classical bisphosphonate drug, can regulate the proliferation and differentiation of osteoblasts and adjust the synthesis of extracellular matrix proteins (ECMs) and bone mineralization [12–14]. The effect of alendronate in the treatment of osteolysis in a rat model was studied by Millett et al. [15] They found that alendronates could effectively prevent peri-prosthetic osteolysis in a rat model. A similar effect has also been found in other animal models, such as canine, mouse, and rabbit models [16, 17]. Zoledronic acid only needs to be intravenously injected once a year, which avoids gastrointestinal side effects and improves the medication compliance of patients [18]. However, zoledronic acid exacerbates inflammation through LPS-induced M1 macrophage polarization, medication-related osteonecrosis often occurs in patients treated with zoledronic acid [19]. Teriparatides (rhPTH1–34) is the only osteogenesis medication approved by the U.S. Food and Drug Administration (FDA) for the treatment of osteoporosis [20]. Teriparatide can stimulate osteoblast activities to increase the formation of cortical and cancellous bone, and can enhance the bio-plasticity of bone [21, 22]. Treatment with teriparatide for 2–4 weeks can increase the bone-prosthesis contact rate in an animal model, as found by Scripitz et al. [23] In addition, teriparatide can significantly inhibit the expression of inflammatory cytokines, such as IL-1 β, IL-6 and TNF α, which are closely related to aseptic loosening [24].

In summary, aseptic loosening of prostheses can be prevented and treated by zoledronic acid and teriparatide, but the specific mechanism and effect still require further research efforts. Based on the characteristics of zoledronic acid and teriparatide, the following hypothesis can be proposed: the combination of the two may have a better treatment effect than the use of either drug alone. No relevant studies have been reported to date. We designed this research and created a rabbit model to study the effect and mechanism of the combination of zoledronic acid and teriparatide in the treatment of aseptic loosening.

## Materials and methods

### Materials

(1) Thirty-two adult male New Zealand white rabbits with a mean weight of 2.75 kg were provided by Shandong Lukang Medicine Corporation (Shandong Province, China). All animal procedures were inspected and approved by the Ethic and Animal W elfare Committee of the Affiliated Hospital of Qingdao University, confirming that all experiments were performed in accordance with relevant guidelines and regulations (Approval Number: N02165782). The study was carried out in compliance with the ARRIVE guidelines.

(2) Titanium rod prostheses were smooth, cylindrical, 2 cm in length, 0.5 cm in diameter, and provided by Stryker Corporation. Autoclaving sterilization was applied before the operation.

(3) Titanium particles less than 20 μm in diameter were provided by Alfa Aesar Chemical Co., Ltd. (China).

(4) Special animal feeding cages and animal experimental operation systems were provided by The Animal Laboratory of The Affiliated Hospital of Qingdao University and assisted by the Medical College of Wisconsin.

(5) Zoledronic acid injection (ZL): (Aclasta, Novartis, Switzerland), 5 mg/100 ml;

teriparatide injection (TP): (Forsteo, Lilly, France), 20 μg/80 μl: 2.4 ml.

### Surgical procedure

**Fig. 1:**
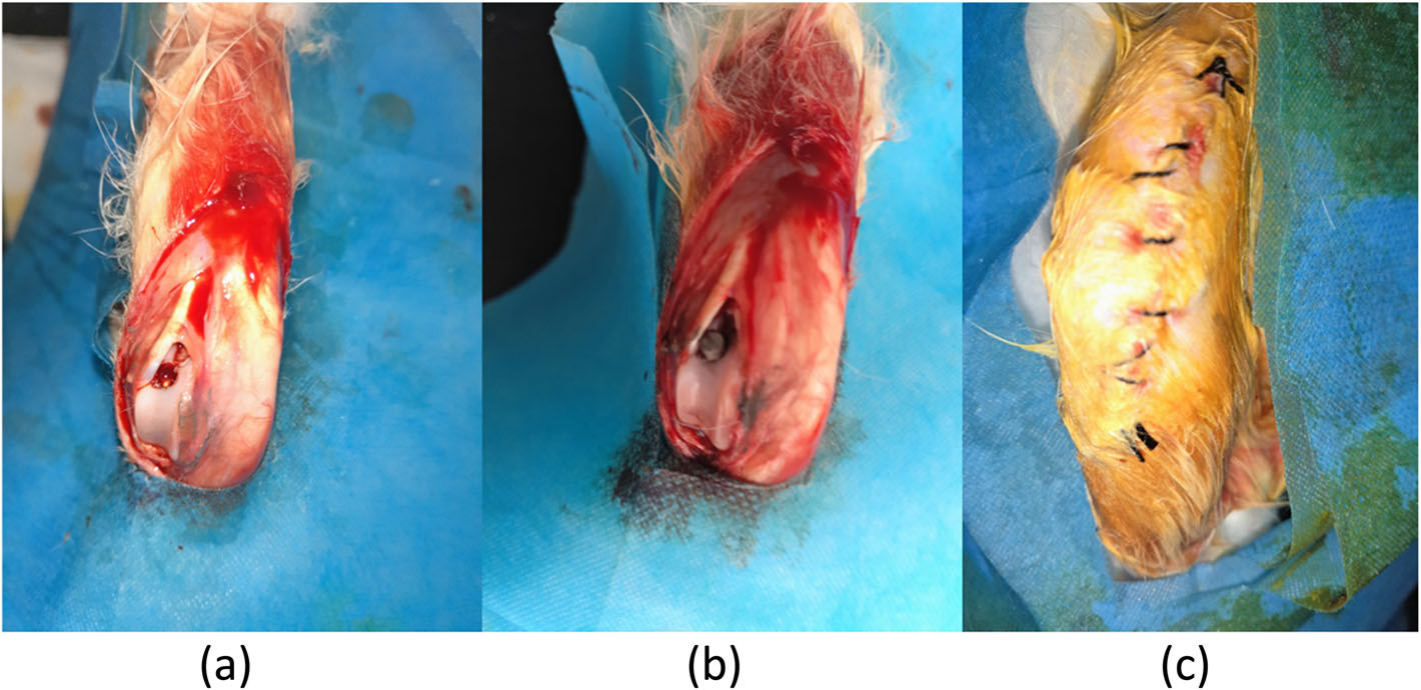
Operative process of titanium rod prosthesis implantation surgery. **a** The medullary cavity was opened along the longitudinal axis of the femur with a 0.3-cm diameter drill at the location of the intercondylar fossa, and then was stepwise expanded with a 0.5-cm diameter drill. **b** The titanium rod implant was implanted in the cavity (depth ≥ 2 cm). **c** The incision was rinsed and closed

The rabbits were adaptively fed for 1 week before the surgery. Rabbits anaesthetized with xylazine hydrochloride (0.2 ml/kg) were fixed in the supine position, the skin of the right knee joint was exposed and sterilized with 2.5% povidone-iodine, and then the surgical area was draped with an aseptic towel with a hole. An anterior midline incision was made in front of the right knee, and the medial parapatellar approach was chosen to expose the knee articular surface. The medullary cavity was opened along the longitudinal axis of the femur with a 0.3-cm diameter drill at the location of the intercondylar fossa, which is above the endpoint of the posterior cruciate ligament, and then was stepwise expanded with a 0.5-cm diameter drill. The titanium rod implant whose surface was evenly coated with approximately 50 μg titanium particles was implanted in the cavity (depth ≥ 2 cm). The incision was rinsed and closed. All surgical procedures were performed under sterile conditions (Fig. 1).

Treatment with 400,000 units of penicillin was performed twice a day to prevent infection after surgery.

### Animal grouping and specimen preparation

Thirty-two rabbits treated with the same surgery and routine treatment were randomized into 4 groups (8 rabbits each). One group was set as the control group (CG), and the other three groups were set according to the postoperative medication: intravenous injection of 0.1 mg/kg zoledronic acid on the second day postoperatively [zoledronic acid (ZL) group]; subcutaneous injection of 20 μg teriparatide, 1 time/day, 5 times/week, continuously for 12 weeks [teriparatide (TP) group]; and intravenous injection of 0.05 mg/kg zoledronic acid on the second day postoperatively + subcutaneous injection of 10 μg teriparatide, 1 time/day, 5 times/week, continuously for 12 weeks [zoledronic acid + teriparatide (ZL + TP) group]. In order to eliminate the effect of doselntensity on the experimental results, the dose of both drugs was halved in ZL + TP group.

Rabbits were sacrificed at 12 weeks postoperatively. The femur and titanium rod were sawed off at a location approximately 2 cm from the knee articular surface. The distal parts of the femur were stored in a refrigerator at − 80 °C, and the cross-sections were made into bone hard tissue sections. Two specimens (approximately 50 μg each) of the residual interface membrane tissue were harvested from the proximal parts of the femur: one specimen was immersed in RNA-later solution, and the other specimen was quickly sealed in a 1.5-ml tube with liquid nitrogen. Both specimens were stored in a refrigerator at − 80 °C for subsequent RT-PCR and Western blotting.

### Radiological evaluation

To observe peri-prosthetic osteolysis and prosthesis loosening, anterior-posterior and lateral right knee X-ray films were performed in all rabbits 2 days before sacrifice.

### Staining and analysis of bone hard tissue sections

The bone-prosthesis tissue was made into undecalcified bone hard tissue sections to investigate bone-prosthesis integration and absorption. The specific steps were as follows:

The 1.0-cm thick bone-prosthesis tissue specimens were made and sliced into 50-μm thick noncontinuous sections along the cross sections of the femur and then stained with Goldner-Masson trichrome staining. The following indicators were observed in the Goldner-Masson trichrome-stained sections:

Bone-prosthesis contact rate (B-PCR, %) = the circumference of the bone contacting the prosthesis/the circumference of the prosthesis.

Bone volume percentage (BVP, %) = bone tissue volume of the 1-mm area around the prosthesis/total volume of the 1-mm area of the prosthesis.

All figures of the hard tissue sections were quantified and calculated with IPP6.0 software.

### Real-time PCR

The expression of mRNA (OCN, OPG, RANKL and TRAP5b) was evaluated by reverse transcription PCR (RT-PCR), and the results were correlated with the differentiation of osteoclasts and osteoblasts.

Total RNA was extracted from the residual interface membrane tissue, and the concentration was tested with a NanoDrop. To acquire cDNA, reverse transcription was performed. The specific steps were as follows: 1) total RNA, 1 μl dNTPs (10 mM each), 1 μl Oligo(DT) _20_ primer (0.5 μg/μl) and DEPC-treated H_2_O were mixed into 12 μl of the reaction mixture, incubated at 65 °C for 50 min and then cooled for at least 1 min; 2) 2 μl 10 × RT buffer, 4 μl MgCl_2_ (25 mM), 2 μl DTT (0.1-M), and 1 μl RNase OUTTM (40 U/μl) and SuperScript III RT (200 U/μl) were added into 8 μl of the main reaction mixture and incubated at 50 °C for 50 min, followed by 85 °C for 5 min, and then were placed on ice; and 3) 1 μl RNase H was added to the main reaction mixture, incubated at 37 °C for 20 min, and then cooled at 4 °C. The primers used in the reaction were designed by Primer5 and synthesized by Invitrogen Co. (Table 1).

**Table 1:** Primer Sequence of target gene and internal reference gene and length of product

Gene name	Primer sequence	Serial number	Product size (bp)
β-actin	Forward:CTGGCACCACACCTTCTACA Reverser:GGTACGACCAGAGGCATACA	NM_007393	446 bp
OCN	Forward:CTGGCTGCGCTCTGTCTCT Reverser:TGCTTGGACATGAAGGCTTTG	NM_010379	198 bp
OPG	Forward:GACAACGTGTGTTCCGGAAA Reverse:TGGTAGGAACAGCAAACCTGAA	NM_008764	399 bp
RANKL	Forward:GCGCAGATGGATCCTAACAGA Reverse:TCTGCGTTTTCATGGAGTCTCA	NM_011613	351 bp
TRAP5b	Forward:GGCCGGCCACTACCCCATCT Reverse:GCCGGCCCCACTCAGCACATAG	NM_007388	178 bp

The cycle number at threshold (CT value) was taken as an internal control, and the expression levels of OCN, OPG, RANKL and TRAP5b in the study groups were compared to those in the control group.

### Western blotting

Western blotting was performed to detect the protein content of OCN, OPG, RANKL and TRAP5b in the interface membrane tissue around the prosthesis. The specimens sealed with liquid nitrogen were homogenized in RIPA buffer (400 μl, Lysis buffer+ Protease inhibitors, at a ratio of 1:100) and then centrifuged at 12000 rpm/min for 10 min at 4 °C. The lysate was collected and denatured in loading buffer (at a ratio of 1:16) and denatured for 10 min at 100 °C. Total protein was harvested and quantified with the BCA™ Protein Quantification Kit, separated by 12% SDS-PAGE and transferred onto PVDF membranes. The membranes were washed in TBST and then immersed in blocking buffer containing 0.05% (v/v) TBST with 5% (w/v) bovine serum albumin (BSA) for 1 h. Primary antibodies [mouse monoclonal anti-OCN (1: 1000 dilution) and anti-RANKL (1:  500 dilution), polyclonal rabbit anti-OPG (1:  500 dilution), anti-TRAP (1:  1000 dilution) and *β*-actin (1:  1000 dilution)] were separately added and placed on a shaker overnight at 4 °C. After washing in TBST, secondary antibodies (HRP conjugated with anti-mouse and anti-rabbit-IgG, 1:  2000 dilution) were separately added and incubated for 1 h at 37 °C. After washing in TBST again, the protein bands were detected by a chemiluminescence detection system. Densitometric analysis was implemented with Quantity One-4.6.5 software.

### Data analysis and statistics

Statistical analyses were performed using SPSS 20.0 and Prism 6.0 software. Data are expressed as the mean ± standard deviation (SD). Differences among groups were analyzed using two-way ANOVA test; subgroup analysis was performed using the LSD test. A value of *p* < 0.05 was considered statistically significant for all analyses.

## Results

### Debris-induced Osteolysis around the prosthesis in radiological examination

**Fig. 2:**
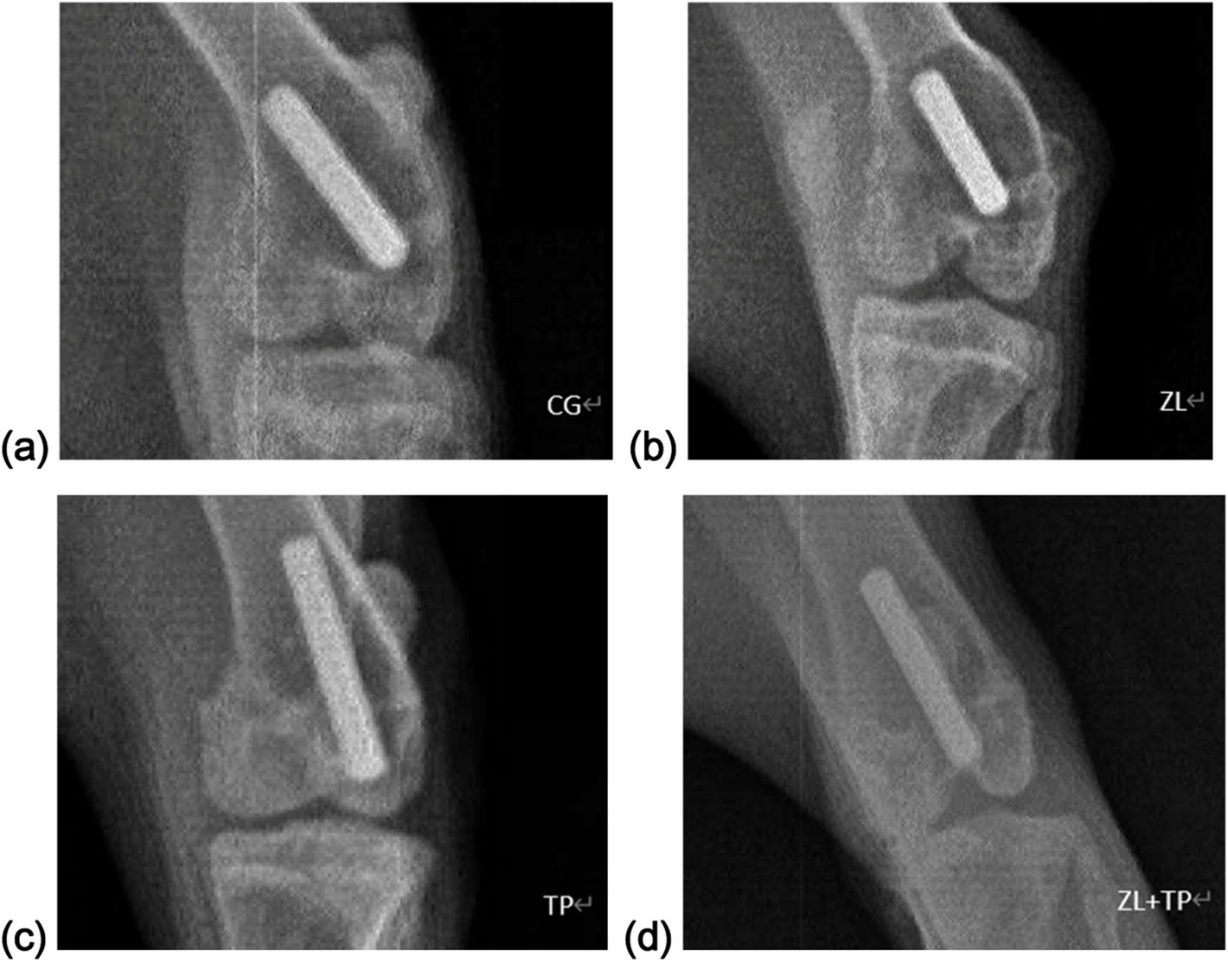
The affected limbs X-ray films of the 4 groups taken at 2 days before sacrifice. Obvious imaging manifestations of peri-prosthetic osteolysis and aseptic loosening that were not detected in the X-ray films of the four groups

The X-ray films of the affected limbs performed 2 days before sacrifice showed obvious imaging manifestations of peri-prosthetic osteolysis and aseptic loosening that were not detected in the X-ray films of the four groups. There were no significant differences in the radiographic examinations (Fig. 2).

### Bone-prosthesis contact in 4 groups

**Fig. 3:**
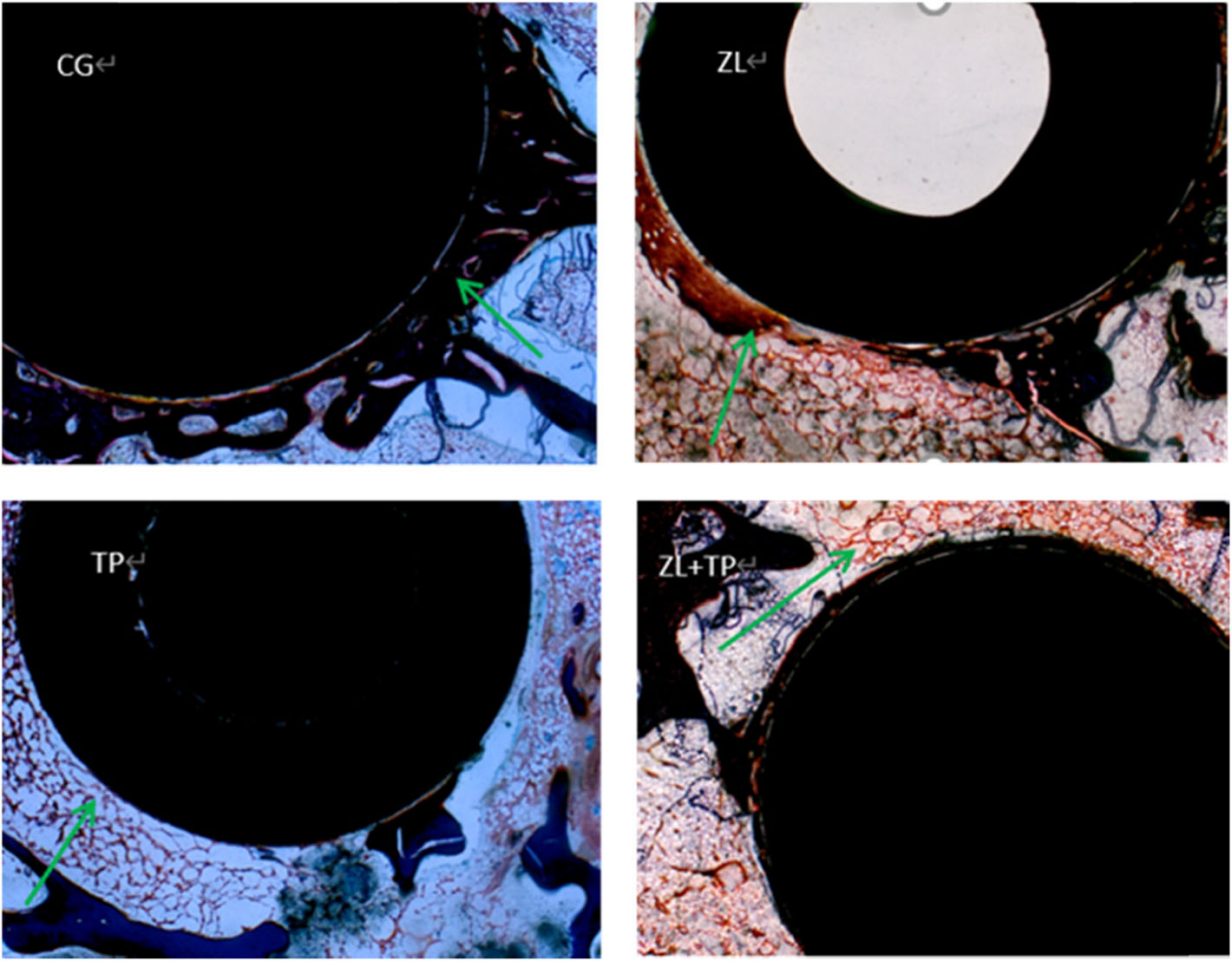
Goldner’s Masson trichrome staining sections of the 4 groups. The bone-prosthesis contact areas are indicated by the green arrows. The integration of the bone and prosthesis in ZL, TP and ZL + TP group was better in the control group. Worse integration of bone and prosthesis and a larger gap could be detected around the prosthesis in the control group

The bone hard tissue sections of the cross sections stained with Goldner-Masson trichrome staining showed that greater bone-prosthesis contact and less fibrous capsule formation around the prosthesis were demonstrated in the ZL group, TP group and ZL + TP group versus the CG group, especially the ZL + TP group (Fig. 3).

**Fig. 4:**
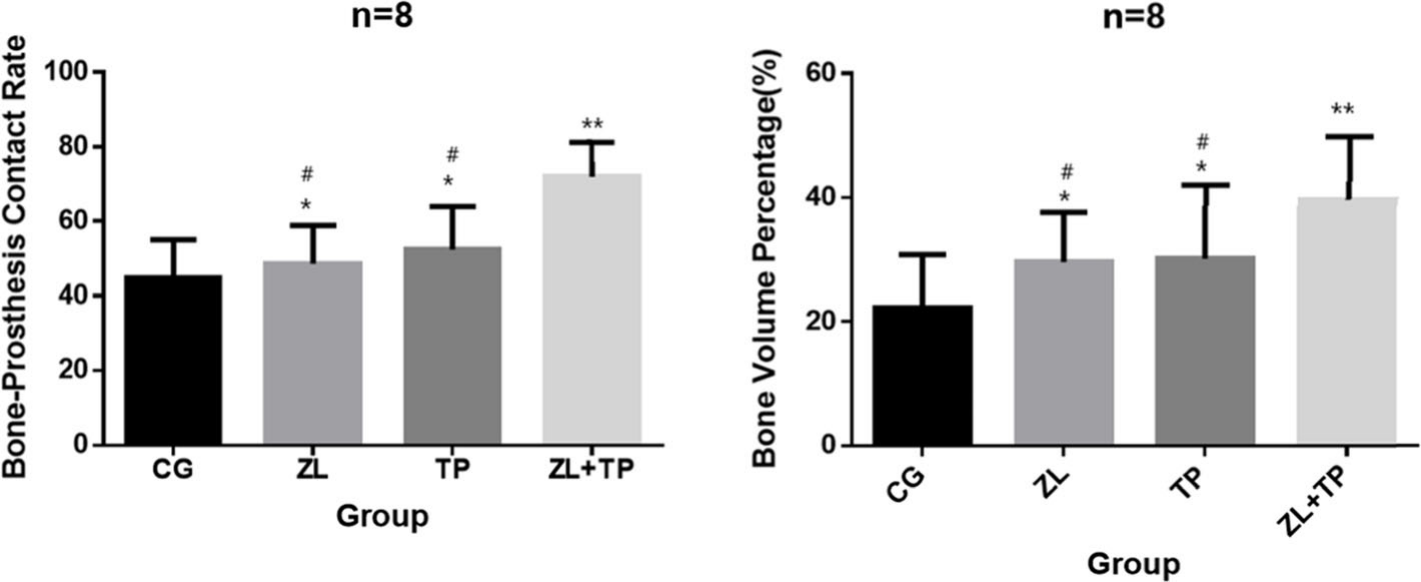
B-PCR and BVF of the 4 groups (x/%, *n* = 16, x ± s). * Significant difference compared to Control group. *P* < 0.05. ** Significant difference compared to Control group. *P* < 0.01. # Significant difference compared to ZL + TP group. *P* < 0.05

The results of Goldner-Masson trichrome staining were analysed by IPP software. The B-PCR (x/%) and BVF (x/%) of the four groups were calculated, and the results of the ZL group, TP group and ZL + TP group were compared with those of the control group. Mild enhancement was present in the ZL group and TP group (*P* < 0.05), but the more apparent enhancement was in the ZL + TP group (Fig. 4), and the difference was statistically significant (*P* < 0.001).

### Higher expression of OCN and OPG and lower expression of TRAP5b and RANKL in the ZL + TP group

**Fig. 5:**
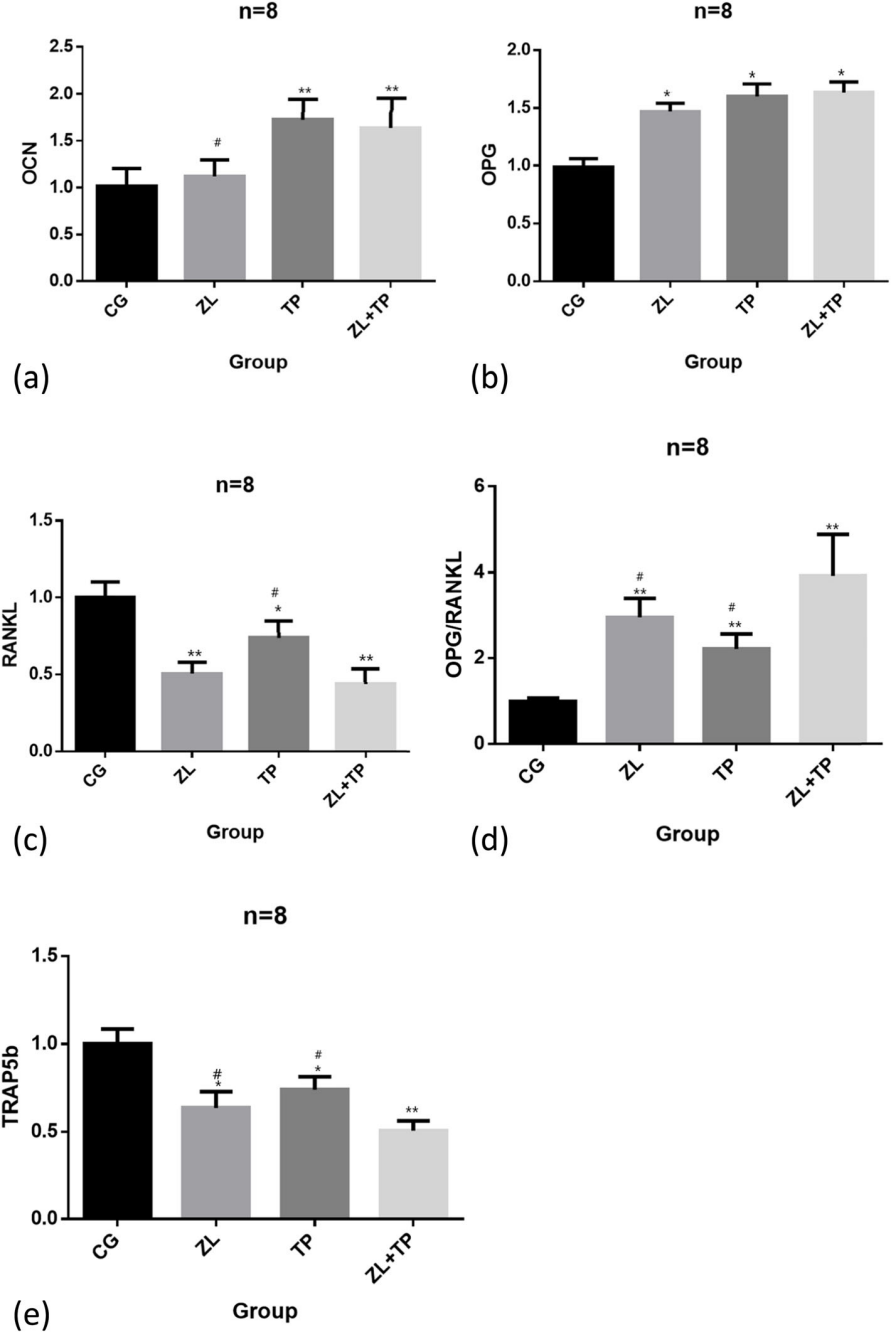
The result of RT-PCR. * Significant difference compared to Control group. *P* < 0.05. ** Significant difference compared to Control group. *P* < 0.01. # Significant difference compared to ZL + TP group. *P* < 0.05

The results of RT-PCR showed that the mRNA content of OCN in the ZL group was not significantly different compared to that in the control group, but in the TP group and the ZL + TP group, it was significantly higher than that in the control group (*P* < 0.01). These results indicated that teriparatide was more potent in stimulating osteoblast activation than zoledronic acid (Fig. 5a).

The mRNA content of OPG in the ZL, TP and ZL + TP groups was higher than that in the control group, while the mRNA content of RANKL in the ZL, TP and ZL + TP groups was lower. The difference was more significant in the ZL + TP group. The OPG/RANGL ratios were significantly higher in the ZL, TP and ZL + TP groups than in the control group and more obvious in the ZL + TP group. This result indicated that ZL + TP combination therapy could inhibit osteoclast activation via the OPG/RANKL pathway, thereby inhibiting osteoclastic bone resorption. The result was statistically significant (*P* < 0.05) (Fig. 5b,c,d).

The mRNA content of TRAP5b around the prosthesis in the ZL, TP and ZL + TP groups was lower than that in the control group, and the difference in the ZL group was more significant than that in the TP group (*P* < 0.05). This result indicated that both ZL and TP could suppress the activation of osteoclasts and that zoledronic acid was more efficacious than teriparatide. The decrease in the mRNA content of TRAP5b in the ZL + TP group was more significant than that in the ZL group and TP group (*P* < 0.05), which indicated that zoledronic acid and teriparatide play a synergistic role in inhibiting osteoclastic bone resorption (Fig. 5e).

**Fig. 6:**
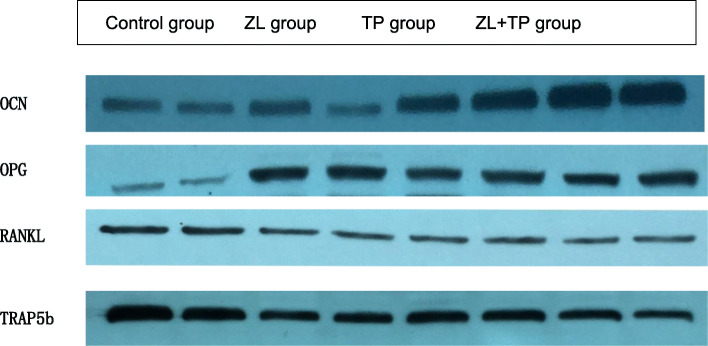
Western Blog testing result. The protein content of OCN in the ZL, TP and ZL + TP groups was significantly higher than that in the control group, while the protein content of TRAP5b was significantly lower than that in the control group. The OPG protein content in the ZL, TP and ZL + TP groups was higher than that in the control group, while the protein content of RANKL was decreased

The results of Western blotting showed that the protein content of OCN in the ZL, TP and ZL + TP groups was significantly higher than that in the control group, while the protein content of TRAP5b was significantly lower than that in the control group. This confirmed that zoledronic acid and tripeptide could stimulate the differentiation of osteoblasts and inhibit osteoclast activation. Regarding the effect on the OPG/RANKL pathway, the OPG protein content in the ZL, TP and ZL + TP groups was higher than that in the control group, while the protein content of RANKL was decreased. This result indicated that osteoclastic bone resorption around the prosthesis was inhibited and that bone formation was increased (Fig. 6).

## Discussion

Particulate debris derived from the prosthesis surface can spread in the articular cavity and adjacent tissues and induce a variety of biological responses, which eventually disrupt the balance of local bone metabolism and result in peri-prosthetic osteolysis and subsequent aseptic loosening. Varieties of pro-inflammatory cytokines can be secreted under the stimulation of particulate debris, such as IL-1α, IL-1β, IL-6, IL-18 and TNF-α. NOD-like receptor protein (NALP3) in the cytoplasm of macrophages can activate the transformation of the IL-1β precursor to the secretory phase and then initiate IL-related inflammatory cascade amplification [25–31]. Macrophages can phagocytize particulate debris ranging from 0.2–10 μm in diameter and release the above pro-inflammatory cytokines that can recruit more macrophages and positively regulate the activation of inflammation [32]. The previous part of this study confirmed that increased RANKL expression and a reduced OPG/RANKL ratio were identified in the articular synovia and peri-prosthesis membranes of patients with aseptic loosening. The imbalance of OPG and RANKL in the bone-growth microenvironment ultimately induced osteoclast activation and local bone resorption [33]. On the other hand, debris-induced pro-inflammatory cytokines can inhibit the functions of osteoblasts to suppress new bone formation and exacerbate peri-prosthetic osteolysis [34].

In view of the above mechanism, aseptic loosening of the prosthesis may be prevented by drugs that can suppress osteoclast differentiation and enhance osteoblast function by acting on related pathways [35–38].

Bisphosphonates can suppress isoprenoid biosynthesis by blocking the mevalonate pathway to induce osteoclast apoptosis and are extensively applied in the treatment of osteoporosis and other metabolic diseases [39]. Additionally, bisphosphonates also target osteoblasts. Treatment with 5 mg zoledronic acid once a year can significantly increase femoral neck bone density by 2.83% [40]. Bisphosphonates may enhance the tensile strength of bone in the process of the treatment of osteoporotic fractures with zoledronic acid [41, 42]. The positive effect of low-dose zoledronic acid on posterolateral lumbar fusion has been observed in rabbit models by Yalcin et al. [43] Zoledronic acid can also effectively prevent peri-prosthetic osteolysis by regulating the OPG/RANKL ratio in animal models [44], and this effect can be achieved by a single postoperative injection of zoledronic acid in mouse models [45]. Even in the presence of wear particles, zoledronic acid can still increase the formation area and thickness of new bone [46]. This study demonstrates that zoledronic acid can increase the bone content around the prosthesis, bone volume fraction and bone-prosthesis contact rate and can also promote bone mineralization around the prosthesis. The mRNA and protein expression of TRAP5b (the specific marker of osteoclasts) decreased significantly, and the OPG/RANKL ratio increased significantly in interface membranes, which indicated that the effect of zoledronic acid in preventing and treating aseptic loosening was achieved by inhibiting the activation and proliferation of osteoclasts and promoting the function of osteoblasts.

The effect of PTH on bone metabolism is related to its concentration. Sustained high levels of PTH can increase osteoclast activity and result in increased bone resorption, and intermittent low levels of PTH can activate osteoblasts and promote bone formation [47]. Many studies have confirmed that teriparatide can increase bone density and reduce the risk of osteoporotic fractures in menopausal women [48]. Low levels of teriparatide can promote fracture healing [49–51], and the sequential treatment of teriparatide and alendronate can sustainedly increase bone density [52]. Osteoblasts play a crucial role in new bone colonization between bone and prosthesis [53]. Bloebaum et al. found that the quantity of osteoblasts in the porous coating-covered area of the prosthesis was significantly higher than that in the non-covered area in animal models, and this phenomenon was significantly reduced on the surface of the eroded prosthesis [54], which confirmed that activation of osteoblasts is an important factor in new bone ingrowth around the prosthesis. Additionally, the early application of teriparatide can increase the thickness of trabecular bone around porous-coated prostheses, as confirmed by Yu XH et al. [55] The non-cemented prosthesis regained stability after 8 months of treatment with teriparatide in the case reported by Zati [56]. Animal experiments have confirmed that teriparatide is superior to alendronate in activating osteoblasts and increasing prosthesis stability and bone mass around the prosthesis [57, 58]. The study also confirmed that the contents of P1NP around the prosthesis were increased significantly after postoperative treatment with teriparatide for 1 year, which resulted in better biological fixation of the prosthesis. This effect was not observed in treatment with alendronate [59]. In this study, the results showed that the B-PCR and BVP in the TP group were both higher than those in the control group and were especially characterized by active bone mineralization. The mRNA and protein expression of OCN in peri-prosthetic tissues was increased compared with that in the control group, which indicated that teriparatide could prevent loosening of the prosthesis by stimulating osteoblast activation to improve osseointegration.

Both zoledronic acid and teriparatide can increase the bone mass around the prosthesis and increase the stability of the prosthesis. The difference is that zoledronic acid mainly inhibits osteoclastic osteolysis around the prosthesis, while teriparatide mainly activates osteoblasts to promote osseointegration and bone formation around the prosthesis. The effects of the combination of zoledronic acid and teriparatide were researched in this study. The B-PCR and BVP in the ZL + TP group were increased significantly, and the efficiency of bone mineralization was also higher than that in the control group. The content of OCN was significantly elevated, which indicated active osteoblast function, while the content of TRAP was significantly decreased, which indicated inhibited osteoclast function. The OPG/RANKL ratio was significantly increased, which confirmed that zoledronic acid and teriparatide play a synergistic role in inhibiting osteoclast activation and stimulating osteoblast activation through the OPG/RANKL pathway, thereby suppressing osteolysis and increasing new bone formation around the prosthesis. According to the above, the effect of the combination of zoledronic acid and teriparatide is superior to the use of either alone in the treatment of aseptic loosening of artificial joints.

The limitation of this study was that the role of mechanical factors in the treatment of aseptic loosening of artificial joints was not confirmed. Second, no adverse reactions of long-term drug use were studied in this experiment. Although the safety and efficacy of the two drugs used alone have been confirmed, there are no related reports of whether the combination of the two drugs will increase the incidence of adverse drug reactions. Third, although the effectiveness of zoledronic acid and teriparatide in the treatment of aseptic loosening was confirmed mechanistically in this study, no evidence of peri-prosthetic osteolysis or bone colonization was observed by imaging. This may be related to the lack of time for inducing aseptic loosening in the animal model or the short time of observation and medication. Extending the observation time should be considered in a follow-up study. Fourth, the effects of zoledronic acid and tripopeptide on inflammatory factors have not been studied, and the role of inflammatory factors in the treatment of aseptic loosening still needs further research. Finally, zoledronic acid and teriparatide are currently more expensive and require a longer period of medication, and the wide application of this combination of medication in the clinic to prevent and treat the aseptic loosening of artificial joints requires more relevant research support.

## Conclusion

This study with a rabbit model of aseptic loosening confirmed that both zoledronic acid and teriparatide could inhibit debris-induced peri-prosthetic osteolysis and promote new bone formation, and this combination can be used in the treatment of aseptic loosening. Zoledronic acid is more capable of inhibiting osteoclast activation and peri-prosthetic osteolysis, while teriparatide is more capable of promoting osteoblast function and peri-prosthetic bone integration. The synergistic combination creates a complementary effect of zoledronic acid and teriparatide to prevent and treat aseptic loosening of prostheses more effectively. However, the safety of the combination and the feasibility of long-term application have not been ensured, and the clinical application requires further experiments and clinical research support.

## Supplementary Information

**Additional file 1: **Figure 6-1 **Western Blog testing result.** The protein content of OCN in the ZL, TP and ZL + TP groups was significantly higher than that in the control group. Figure 6-2 **Western Blog testing result.** The protein content of OPN in the ZL, TP and ZL + TP groups was higher than that in the control group. Figure 6-3 **Western Blog testing result.** The protein content of RANKL in the ZL, TP and ZL + TP groups was significantly higher than that in the control group. Figure 6-4 **Western Blog testing result.** The protein content of TRAP5b in the ZL, TP and ZL + TP groups was significantly lower than that in the control group.

**Additional file 2.**

**Additional file 3.**

**Additional file 4.**

**Additional file 5.**

## Data Availability

All data concerning the resarche are presented in the manuscript.
