# Supplementary material for: Zoledronic acid and teriparatide have a complementary therapeutic effect on aseptic loosening in a rabbit model
Source: BMC Musculoskelet Disord. 2021 Jun 24;22:580. doi: 10.1186/s12891-021-04458-4 (PMC8223324; doi:10.1186/s12891-021-04458-4)
Supplement: Supplementary file 1 — Additional file 1: Figure 6-1 Western Blog testing result. The protein content of OCN in the ZL, TP and ZL + TP groups was significantly higher than that in the control group. Figure 6-2 Western Blog testing result. The protein content of OPN in the ZL, TP and ZL + TP groups was higher than that in the control group. Figure 6-3 Western Blog testing result. The protein content of RANKL in the ZL, TP and ZL + TP groups was significantly higher than that in the control group. Figure 6-4 Western Blog testing result. The protein content of TRAP5b in the ZL, TP and ZL + TP groups was significantly lower than that in the control group. [file 12891_2021_4458_MOESM1_ESM.docx]

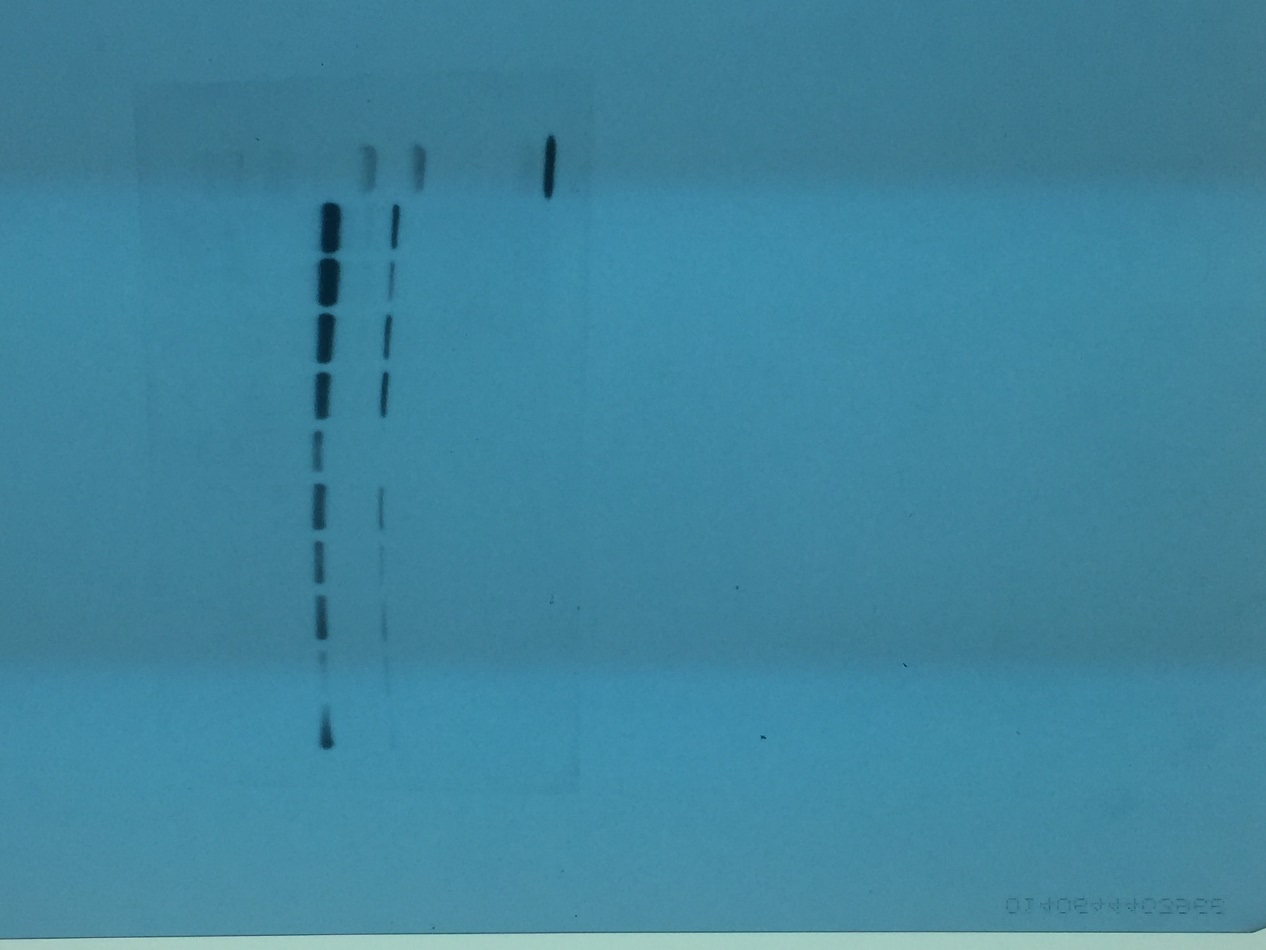


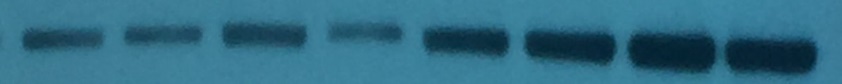


Figure6-1 **Western Blog testing result.** the protein content of OCN in the ZL, TP and ZL+TP groups was significantly higher than that in the control group
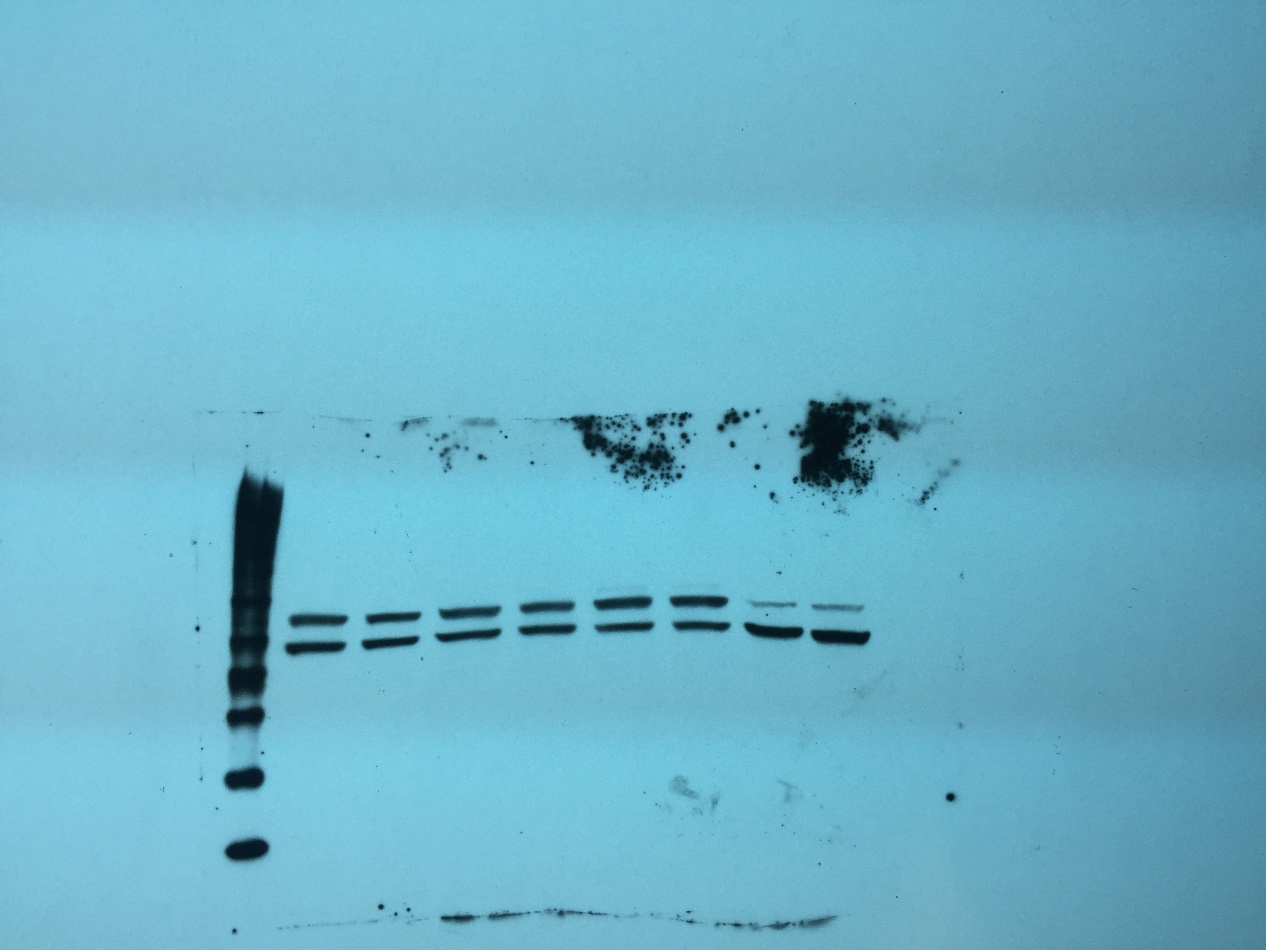


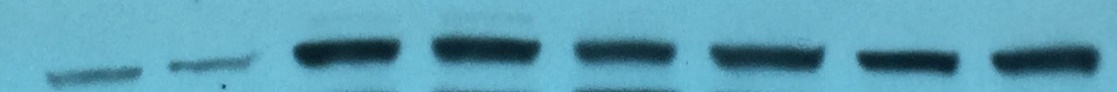


Figure6-2 **Western Blog testing result.** the protein content of OPN in the ZL, TP and ZL+TP groups was higher than that in the control group
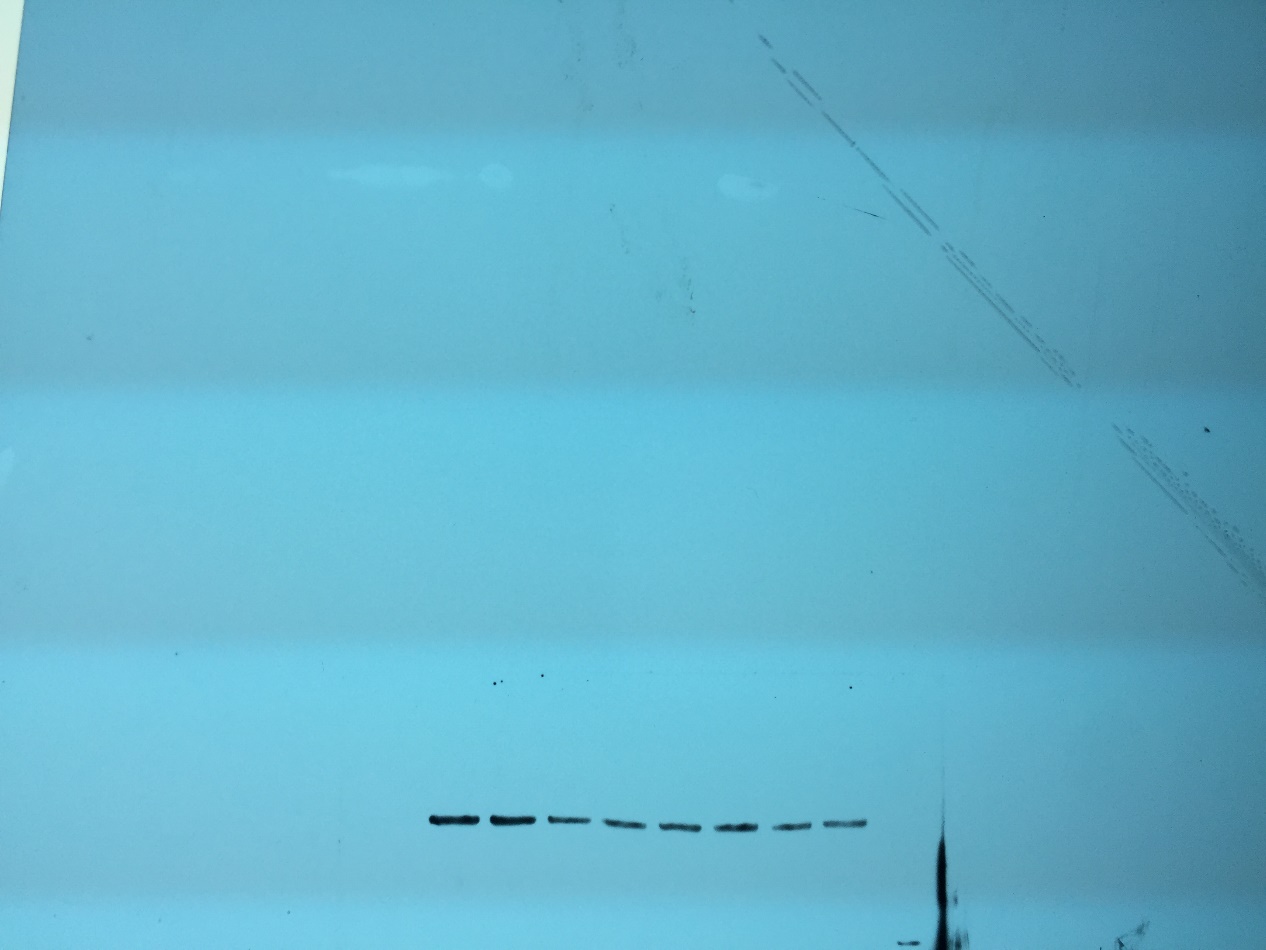


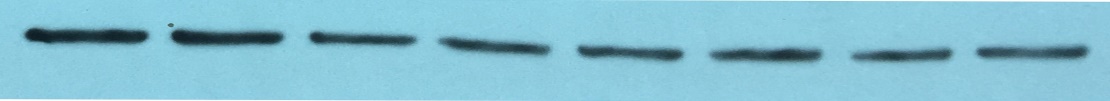


Figure6-3 **Western Blog testing result.**

the protein content of RANKL in the ZL, TP and ZL+TP groups was significantly higher than that in the control group
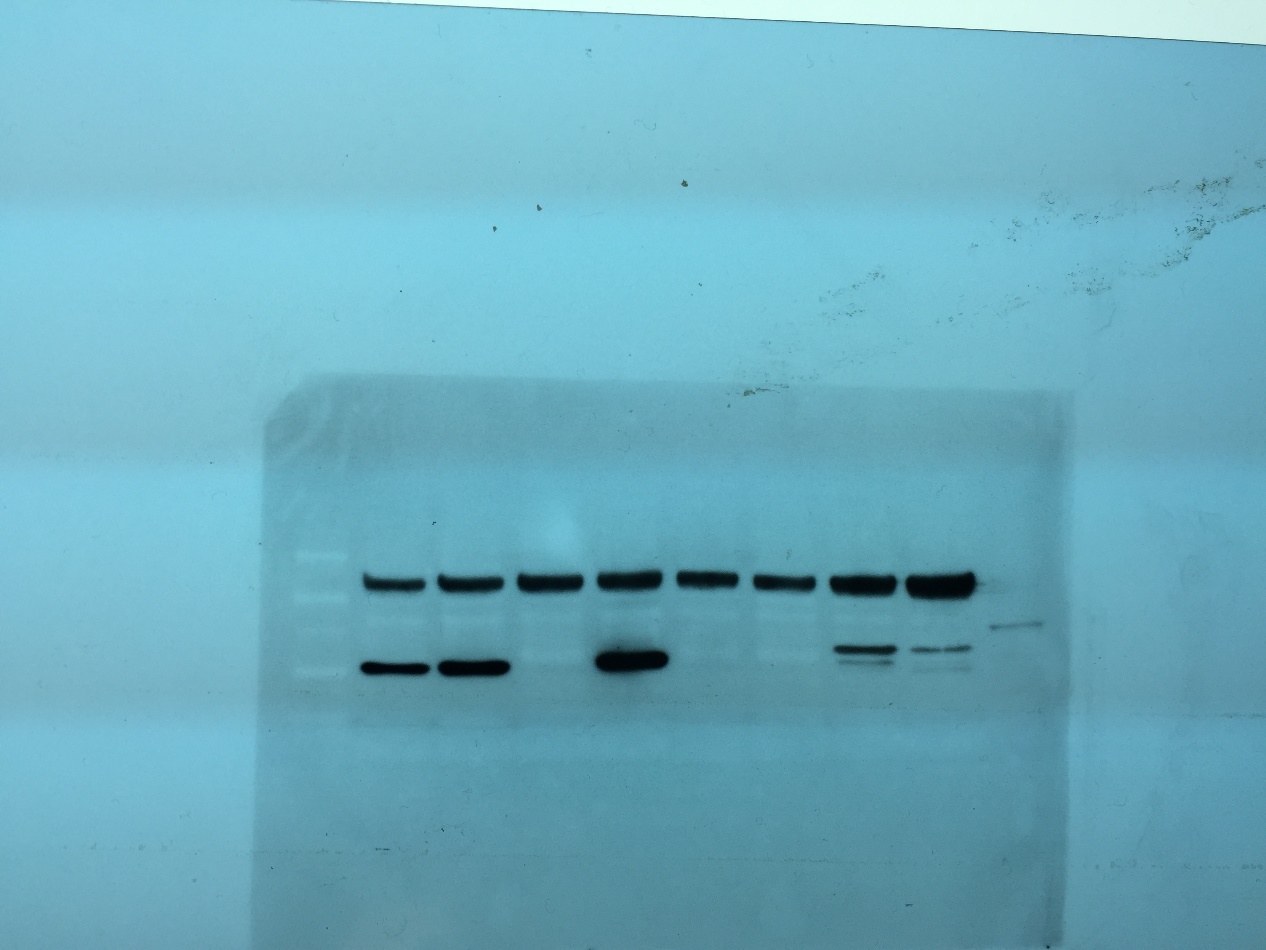


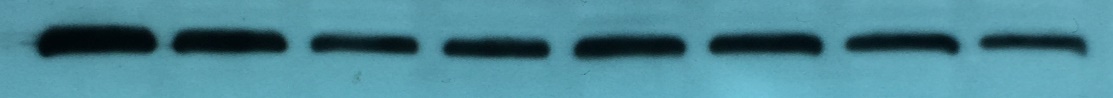


Figure6-4 **Western Blog testing result.**

the protein content of TRAP5b in the ZL, TP and ZL+TP groups was significantly lower than that in the control group.
