# Supplementary figures and images for: Zoledronic acid and teriparatide have a complementary therapeutic effect on aseptic loosening in a rabbit model
Source: BMC Musculoskelet Disord. 2021 Jun 24;22:580. doi: 10.1186/s12891-021-04458-4 (PMC8223324; doi:10.1186/s12891-021-04458-4)

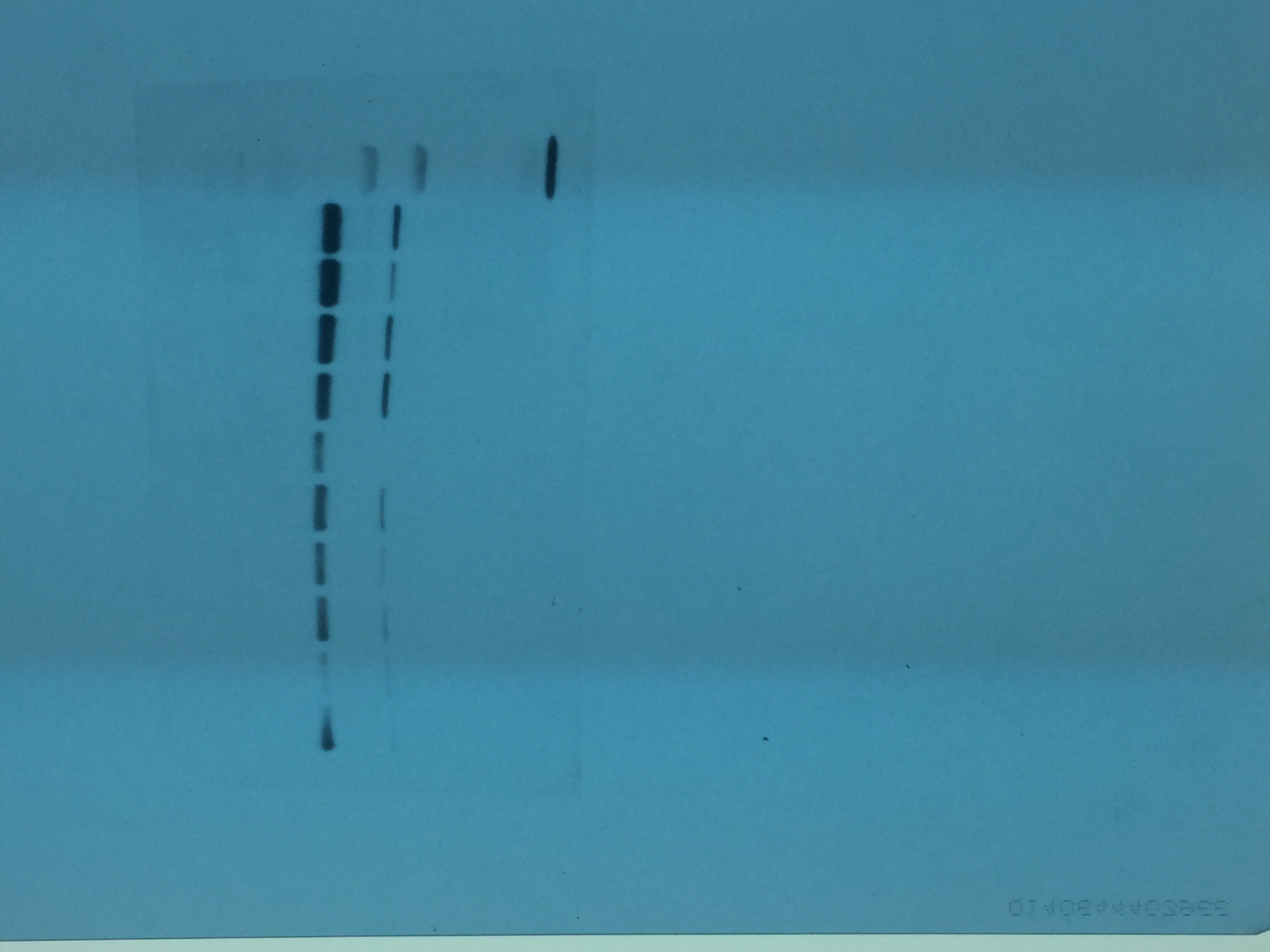

Supplement: Supplementary file 2 — Additional file 2. [file 12891_2021_4458_MOESM2_ESM.jpg]

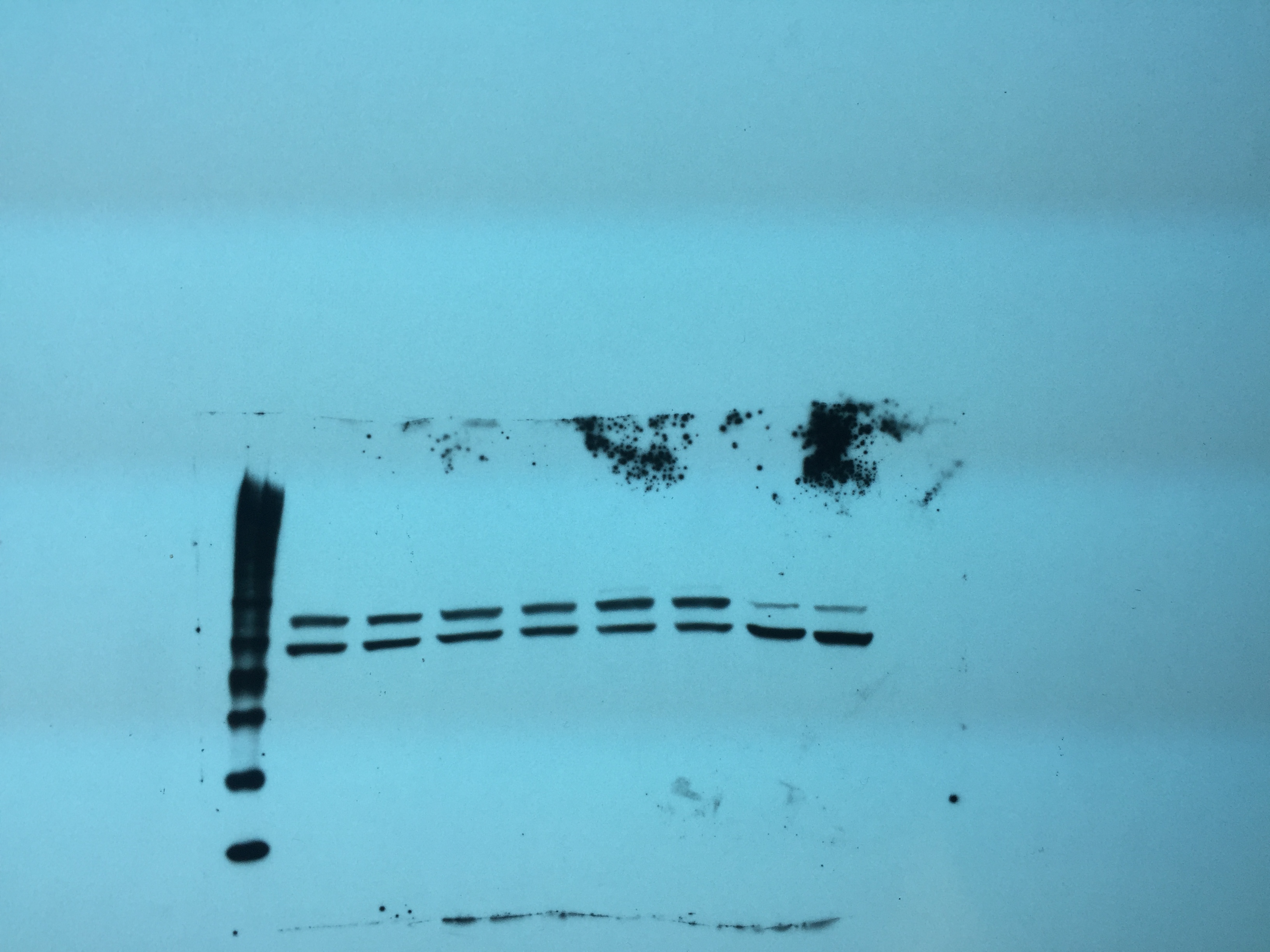

Supplement: Supplementary file 3 — Additional file 3. [file 12891_2021_4458_MOESM3_ESM.jpg]

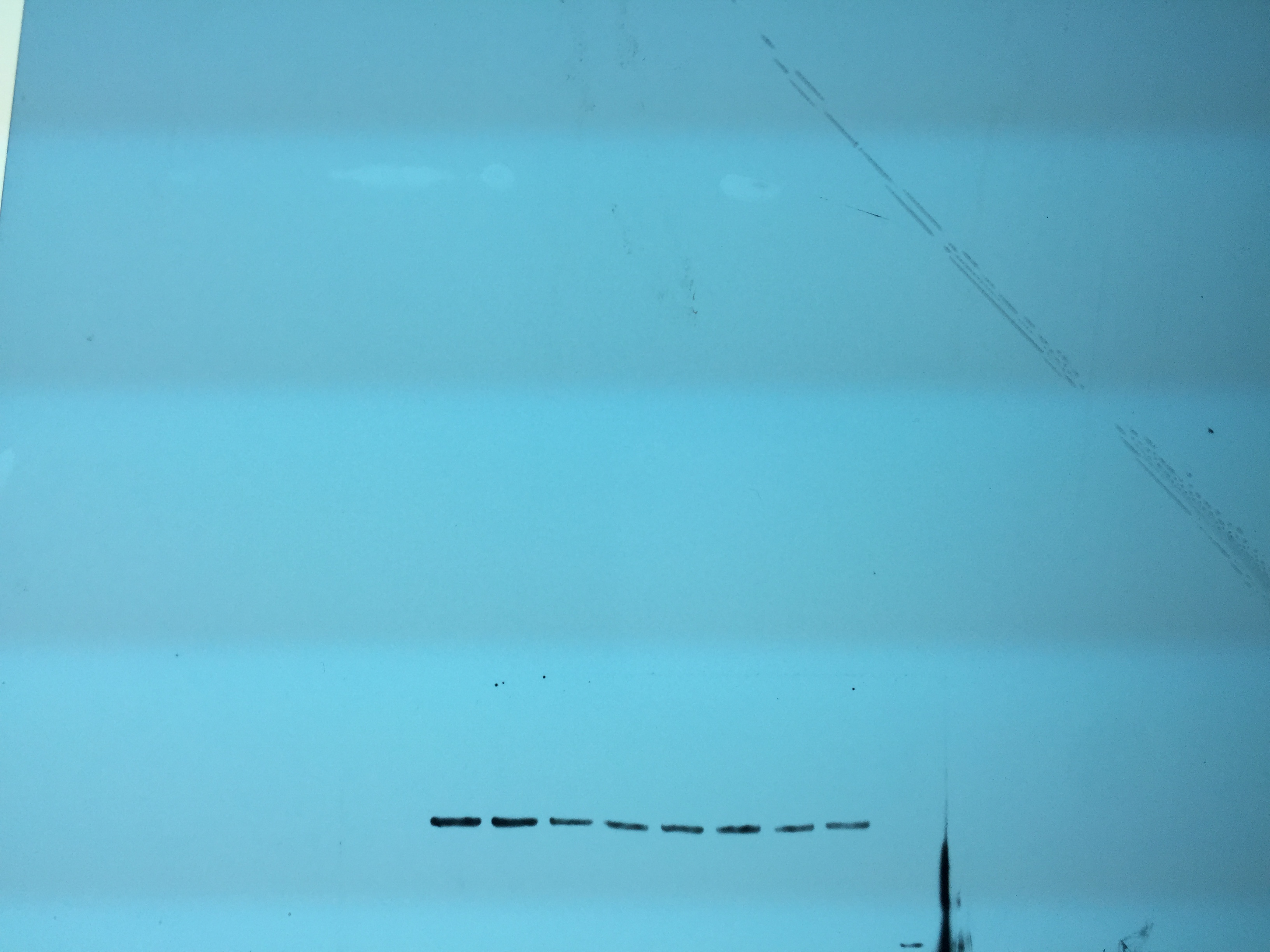

Supplement: Supplementary file 4 — Additional file 4. [file 12891_2021_4458_MOESM4_ESM.jpg]

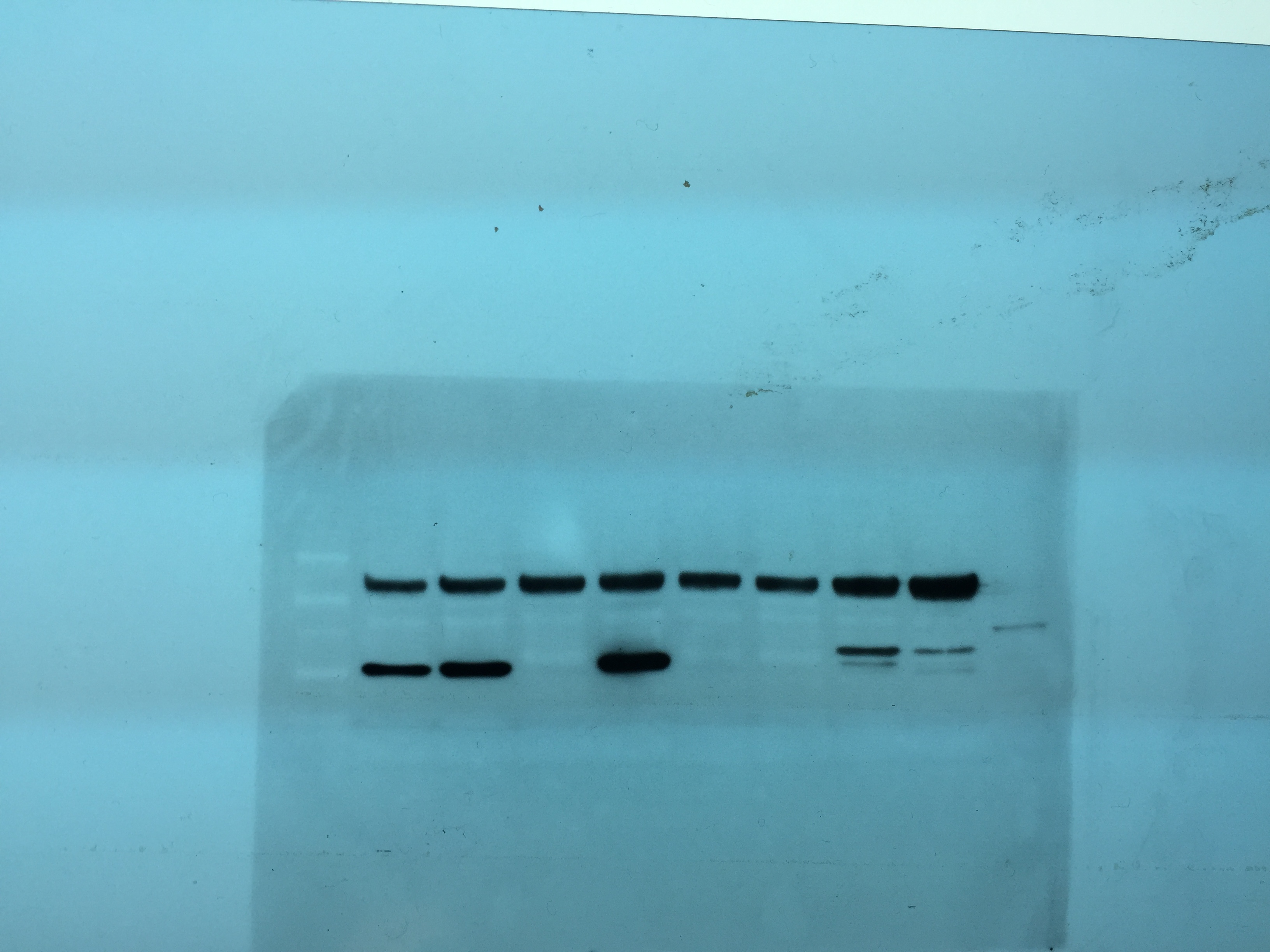

Supplement: Supplementary file 5 — Additional file 5. [file 12891_2021_4458_MOESM5_ESM.jpg]
